# School closure as an influenza mitigation strategy: how variations in legal authority and plan criteria can alter the impact

**DOI:** 10.1186/1471-2458-12-977

**Published:** 2012-11-14

**Authors:** Margaret A Potter, Shawn T Brown, Phillip C Cooley, Patricia M Sweeney, Tina B Hershey, Sherrianne M Gleason, Bruce Y Lee, Christopher R Keane, John Grefenstette, Donald S Burke

**Affiliations:** 1 Graduate School of Public Health, University of Pittsburgh, Pittsburgh, Pennsylvania, USA; 2 Pittsburgh Supercomputing Center, Pittsburgh, Pennsylvania, USA; 3 RTI International, Research Triangle Park, Durham, North Carolina, USA; 4 School of Medicine, University of Pittsburgh, Pittsburgh, Pennsylvania, USA

## Abstract

**Background:**

States’ pandemic influenza plans and school closure statutes are intended to guide state and local officials, but most faced a great deal of uncertainty during the 2009 influenza H1N1 epidemic. Questions remained about whether, when, and for how long to close schools and about which agencies and officials had legal authority over school closures.

**Methods:**

This study began with analysis of states’ school-closure statutes and pandemic influenza plans to identify the variations among them. An agent-based model of one state was used to represent as constants a population’s demographics, commuting patterns, work and school attendance, and community mixing patterns while repeated simulations explored the effects of variations in school closure authority, duration, closure thresholds, and reopening criteria.

**Results:**

The results show no basis on which to justify statewide rather than school-specific or community-specific authority for school closures. Nor do these simulations offer evidence to require school closures promptly at the earliest stage of an epidemic. More important are criteria based on monitoring of local case incidence and on authority to sustain closure periods sufficiently to achieve epidemic mitigation.

**Conclusions:**

This agent-based simulation suggests several ways to improve statutes and influenza plans. First, school closure should remain available to state and local authorities as an influenza mitigation strategy. Second, influenza plans need not necessarily specify the threshold for school closures but should clearly define provisions for early and ongoing local monitoring. Finally, school closure authority may be exercised at the statewide or local level, so long as decisions are informed by monitoring incidence in local communities and schools.

## Background

School closure has long been considered a useful social-distancing strategy to control the spread of infectious diseases among children [1-4], who are efficient transmitters of influenza virus [5,6] and whose immunity to circulating virus strains may be lower than that of adults [7]. School closures may delay an epidemic peak, allowing more time for vaccine distribution and readying the healthcare system (*e.g.*, increasing available beds) [8].

Nevertheless, state and local officials faced uncertainty during the 2009 influenza H1N1 epidemic about whether, when, and for how long to close schools. Between April and May, the World Health Organization and the U.S. Centers for Disease Control and Prevention changed their advice from recommending closures unequivocally to deferring to state and local decision makers [9,10]. But there was inconsistency as to which agencies and officials had legal authority over school closures [11]. Moreover, the evidence base for when and how long to close schools was sparse [12]. Though ordered for some schools in spring, summer, and fall of 2009, school-closures were determined typically by local school districts and individual schools; and closure durations rarely exceeded a few days [13,14]. It was reported that H1N1 school closure experience had much local variation in decision-making authority, disagreement between school administrators and public health officials, and lack of clarity about the goals for closure [15]. A review of laboratory-confirmed H1N1 cases in Alberta, Canada found that the 2009 summertime closure of schools had interrupted virus transmission among school-aged children but then failed to prevent a second wave of the pandemic when schools re-opened in the fall [16].

The relatively mild transmissibility and severity of the 2009 H1N1 disease contrasts with the reasonable expectation that another more virulent novel pandemic disease will occur in the future—one accompanied by surging demand for healthcare services, lack of a vaccine, and high morbidity and mortality rates. The need remains to augment the evidence base and to plan meaningfully for school closures.

First, should school closure authority as an influenza mitigation strategy be centralized statewide or decentralized locally? A single, statewide official with recognized authority over all school districts and localities might have greater effect in closing schools uniformly across a state; whereas distributed authority among officials or decentralized authority structure might cause delay or non-uniformity in the timing of school closures decisions and implementation. The effects of these policy alternatives on the spread of infection are unknown.

Second, how early—that is, at what prevalence threshold—should schools close to mitigate a pandemic? School closures are costly [17-19], burdensome on workers and families [20], and a potential cause of absenteeism among public health workers [21]. Caution in imposing such economic and social burdens must be balanced against the health benefits; but neither past experience nor empirical study has yet suggested sound metrics to guide such decision-making.

Third, for how long should schools remain closed? A previous study showed that increasing the duration of school closures reduced the attack rate of infections in a county population and that limiting the duration to two weeks or less—when virus was still circulating—was associated with increased attack rates [8]. Without empirical evidence, the high costs of school closure could exert pressure on school districts, individual schools, and geographic localities to reopen too soon.

Establishing an evidence base for pandemic planning is challenging, since school closure alternatives cannot be tested in controlled observational studies, experience with one influenza outbreak cannot necessarily be generalized to others, and isolating school closure as a mitigation strategy from other social-distancing or pharmaceutical interventions is infeasible in practice. The present study avoided the limitations of observational studies by using a large-scale computational model to provide insights and guidance for policy makers. Here, we conducted repeated simulations of epidemics with specifications and assumptions held constant while varying school closure policies and observing the outcomes.

Previous observational studies have analyzed epidemic patterns and outcomes from actual experience in which school closures were either coincident with a holiday or vacation period [6]. Here, the purpose is to test the epidemic implications of criterion-based school closure decision making. Therefore, the simulations conducted in this study use a pro-active approach to closures, geared to the actual variations of authority and specifications found in state statutes and pandemic influenza plans.

## Methods

This study began with analysis of school-closure statutes and published pandemic influenza plans in all fifty U.S. states and interpreted the variations among them for application to modeling experiments. We chose to use the population dynamics of one state (Pennsylvania) to represent constants for demographics, commuting patterns, work and school attendance, and community mixing patterns while repeated simulations explored variations in delegation of school closure authority, closure duration, closure thresholds, and reopening criteria. Epidemic simulations using a large-scale agent-based model explored whether and how the variations might affect outcomes as measured by attack rate, peak pandemic day, and peak case incidence.

Throughout this paper, we use the terms “pandemic” and “epidemic” to denote separate meanings. “Pandemic” refers to the H1N1 outbreak of 2009–2010 that was officially declared as such and describes the state plans so labeled. “Epidemic” describes the results of the simulations produced by our agent-based model.

### School closure statutes and pandemic influenza plans

An analysis of states’ school closure statutes and pandemic influenza plans identified variations influencing the uniformity, timing, and duration of closures that lead to a typology of school-closure authorities and criteria.

First, we re-analyzed data from a previously published report [11] of school closure statutes to identify characteristic ways of delegating school closure authority among states. Statutes delegated a single state official (14 states), one state official and one per-locality official (14 states), a single per-locality official (15 states), or two or more state officials and/or two or more per-locality officials (4 states); there was no statutory designation of authority in 3 states. These variations could affect whether or not a school-closure order could be implemented uniformly throughout a state and whether disagreements among designated officials might cause delays in implementation.

Second, we analyzed states’ pandemic influenza plans to identify criteria for initiating and terminating school closures. Every state had developed such a plan under direction of the U.S. Department of Health and Human Services (DHHS) during the avian influenza alert of 2005 and again in 2008. In the spring of 2010, we downloaded and analyzed the plans then posted on the DHHS website and followed links to state websites for further information. When links were broken, we added Google searches using the search phrase “[state name] pandemic influenza plan,” which led to the specified documents. These methods produced complete plans for 45 states and plan summaries for 3 states (Iowa, North Dakota and Washington) but no plan for 2 states (Massachusetts and Rhode Island); so our analysis used only 48 state plans. These were dated from 2005 to 2010, with the majority written in 2006. All plan documents used in this analysis and downloaded in portable document format (pdf) are maintained on our study’s website [22].

Almost all the plans discussed school closure as an influenza mitigation strategy. Closure criteria were either stated as incidence at the state-wide or community-wide level (35 states), indicating uncertainty as to whether statewide uniformity or local autonomy is likely to be more effective in mitigating the spread of infection. However, plans rarely stated a specific incidence level to trigger closures, probably reflecting the lack of evidence to guide such advice. No closure criteria were mentioned in 14 state plans. State plans had criteria for re-opening schools either in-depth (13 states), or briefly (8 states), or not at all (28 states). Again, the lack of specificity is attributable to the lack of empirical evidence; however, without such criteria for disease control, official decision makers are left subject to economic and social pressures to re-open schools from parents and employers.

Several examples illustrate how state planners have dealt with the lack of empirical evidence to guide school closure and re-opening thresholds. The Tennessee plan states that schools are to be closed when three conditions are met: 1) pandemic virus causing morbidity and mortality in excess of seasonal influenza; 2) laboratory confirmation of the pandemic virus in a county or surrounding county; and 3) state surveillance indicating community spread of the pandemic virus in the county or a surrounding county. This plan specifies that schools should re-open based on statewide criteria: when the State Epidemiologist determines that “the pandemic wave has subsided” based on sentinel surveillance. The Kentucky plan instructs decision makers to "Collaborate with the local school board for closing and re-opening of school” [22]. The North Carolina plan asserts that “No data exists *sic* for recommending illness thresholds or rates of change in number of illnesses that should lead to consideration of dismissing or reopening schools”.

### Interpretation of state variations for agent-based model

The typical variations among statutory authorities and the uncertainties reflected in state plans are likely to affect school closures in terms of statewide uniformity, timing in relation to disease prevalence, and overall duration. Further, these variations are likely to produce differences in outcome of an epidemic, including attack rate (infected and symptomatic individuals as a proportion of total population), peak epidemic day, and peak case incidence (number of cases by day). These are outcomes that affect demand for healthcare services overall as well as hospitalizations and mortality.

For the purpose of our simulations, the observed variations in states’ statutes and pandemic plans are captured in five “types”:

Type I represents uniform statewide school closure order based on a standard threshold of statewide prevalence with all schools implementing closure within 1 day and remaining closed for the specified duration. We tested 1% prevalence for alternative closure durations of 1, 2, 4, 8, and 16 weeks; we also tested 0.1% and 10% prevalence for the closure duration of 8 weeks.

Type II represents a local threshold for school closure based on a standard of 5 cases in any school at alternative closure durations of 1, 2, 4, 8, and 16 weeks.

Type III is similar to Type I but varies the period of implementation for a statewide closure order, testing alternative implementation delays of 3, 5, and 10 days. Such delays could account for time needed to resolve disagreements among state and/or local officials holding concurrent authority for decision making.

Type IV represents lack of statewide uniformity by introducing randomness in the threshold for closures at the individual school level: 1 to 3 cases, 1 to 5 cases, and 1 to 10 cases. For example, in the 1–3 case scenario, each school in the model has a random chance of closing at either 1, 2, or 3 days after a closure order; in the 1–5 case scenario, each school has a random chance of closing at 1, 2, 3, 4, or 5 days after a closure order. For all scenarios each school, once closed, remains closed for the specified duration.

Type V represents how local pressures might limit the duration of closures by randomly re-opening schools before the specified duration. Prematurity alternatives of 1–3 days, 1–10 days, 1–20 days, and 1–30 days were tested. Randomness in each alternative scenario determined when any particular school would re-open.

### Agent-based model and epidemic simulation

An agent-based model represented each individual person living in the state of Pennsylvania and was similar in design to previously described models of Allegheny County, Pennsylvania [8,23], and the Washington, DC metropolitan area [24,25]. The complete list of data sources appears in Table 1.

**Table 1:** Data Sources for Pennsylvania School Closure Model

**Specification/Calibration**	**Data Source**
Pennsylvania population, with distributions by age, sex, employment status, and household location	PA model represents a population of 11,863,395 people (This excludes populations that reside in close group quarters such as prisons)
	Method to extract the agent population from Census data was developed by Beckman et al. [26].
	Pennsylvania data from US Census Bureau’s Public Use Microdata files and Census aggregated data [27].
Pennsylvania location specifications	316,148 workplaces (ESRI Business Analyst GIS data product)
	4,319 schools (National Center for Education Statistics [28])
	4,779,182 households (Pennsylvania data from US Census Bureau’s Public Use Microdata files and Census aggregated data [27])
Students assigned to schools	Overall methodology described [29].
	Pennsylvania data on public and private schools and school assignments (National Center for Education Statistics [28])
Employed adults assigned employment locations	Pennsylvania data on workplaces [30] and workplace assignments were taken from US Census Standard Tabulation Product (STP64) and ESRI Business Analyst GIS respectively.
Transmission site assumptions for homes, schools, worksites, and communities	Calibrate to a pandemic of R_0_ = 1.4 (approximately 34% of population has symptomatic illness) with 33%, 12.5%, 24.5% and 30% of transmissions occurring in the household, workplace, schools and community respectively [31].
	Natural history parameters for transmission probabilities under varying conditions are given in Table 1 of reference [31].
	R_0_ = 1.2, 1.6, and 2.0 were simulated by scaling the transmissibility of the disease to produce approximately 19%, 36% and 45% symptomatic illness in the population respectively.

A geospatially explicit human agent database, termed a synthetic population, represented the state of Pennsylvania in the year 2000. Each agent was assigned to a household, so that at the census tract level the synthetic population contained realistic distributions of households, and agent demographics. Thus, the model depicted the region’s individual households, schools (K-12), workplaces, and healthcare facilities, with agents assigned to each using previously described methods and the following data sources: schools and school assignments—U.S. Department of Education National Center for Education Statistics (public schools data) and private data vendor (private schools); workplaces and workplace assignments—U.S. Census Standard Tabulation Product (STP64) commuting pattern data and ESRI Business Analyst (InfoUSA business data).

It is assumed that when the epidemic starts, schools are open. On weekends, students do not go to school and instead have increased activity in their neighborhoods and communities. For each scenario, the results presented are the average of 20 stochastic simulation runs, a number sufficient to obtain statistically significant results from computed confidence intervals. Each simulation weekday, agents moved among their respective households, their assigned workplaces (or schools depending on their age), and various locations in the community, where they interacted with other proximal agents based on the rates in Table 2. Agents interacted more frequently with agents with whom they had close relationships (e.g., family members, household members, classmates, and office mates). Employees of large firms interacted more closely with their office mates but also encountered people working in different offices of the same firm. Workers in firms having only one office repeatedly contact the same people each day.

**Table 2:** **Model Transmission and Person-to-Person Contact Parameter Values**[32]

**Contact Group**	**Infected**	**Susceptible**	**Value**
Household	Adult	Adult	.4
Household	Child	Adult	.3
Household	Adult	Child	.3
Household	Child	Child	.6
School	Elementary Student	Elementary Student	.0435
School	Middle Student	Middle Student	.0375
School	High Student	High Student	.0315
Workplace	Adult	Adult	.0575
Hospital	HCW	HCW	.0575
Hospital	HCW	Patient	.01
Hospital	Patient	HCW	.01
Neighborhood	All	Child	.0000145
Neighborhood	All	Adult	.000725
Community	All	Child	.00003175
Community	All	Adult	.00018125

The model implements school closures by halting the contacts occurring when schools are open. During closures, alternative contact patterns are implemented, as specified above.

### Disease parameters and model calibration

An underlying Susceptible- Exposed-Infectious-Recovered (SEIR) disease model governed disease progression and transmission. At the start of each simulation run, all people are susceptible (S) to influenza, based on the assumption of an entirely novel virus. On Simulation Day 1, introduction of 100 infectious individuals into random locations triggers the epidemic. Every susceptible individual who contacts an infectious individual has a probability of contracting influenza (Table 2) derived from prior studies of the 1957–8 Asian influenza pandemic. Each newly infected person then moves to the exposed (E) state for the duration of the disease's incubation period and then to the infectious state (I) where the person could infect others. One-half of infectious patients exhibit symptoms, 50% of sick students and workers stay home with no community contacts unless they see a doctor, and 40% of symptomatic patients visit a clinic or emergency room. Following the infectious period, the agent proceeds to the recovered state (R), in which he or she is immune to subsequent infections. Initial calibration of this model utilized the Ferguson *et al.* approach with data from historical (1957–58, 1968–69) influenza pandemics and targeted an epidemic with a 35% attack rate seen in the 1957–58 pandemic [33-36].

The mitigating effect of the weekend on influenza transmission has been widely reported [37,38]. For example, the study by Hens *et al.* in eight European countries estimated a 10 ~ 20% reduction in influenza infections during weekend when compared to weekdays [37]. A primary reason is that most workplaces and schools are closed simultaneously during the weekend, and thus fewer human contacts take place as opposed to weekdays. For instance, a survey by McCaw *et al.* indicated that an individual has 2 ~ 4 more personal contacts during weekend than weekdays [39].

Cauchemez et al. [40] in a study of influenza spread in France showed that extended holidays result in a 20–29% reduction in the rate influenza as transmitted to children but have no detectable effect on the contact patterns of adults. Holidays prevent 16–18% of seasonal influenza cases (18–21% in children). By extrapolation, we find that prolonged school closure during an epidemic might reduce the cumulative number of cases by 13–17% (18–23% in children) and peak attack rates by up to 39–45% (47–52% in children). Of course this impact would be reduced if the reduced contact rates among children cannot be maintained for a prolonged period.

With respect to workers, the US labor force of 154 million people as of August 2009 spent an average of 7.9 hours per weekday and 5.6 hours per weekend day working, presumably interacting closely with each other and clients [41].

For our model on weekends, schools and many workplaces are closed. We assume that, then, 50% of student agents increase their community interactions by 50% for an average of 25% increase which approximates those in [39]. A minority (20%) of employees continued to work and maintain their normal weekday level contacts on weekends. We also assumed that 50% of sick students and workers stayed at home and did not interact with anyone outside of the household. Our workplace absentee rate is consistent with other models but slightly higher than published employee absenteeism estimates during an influenza epidemic that range from 10 to 40 percent (see Thanner et al. [42]). However, we used a school absentee rate that is lower than some models (Ferguson et al. [D] use a 90% absentee rate), but higher than those reported during the 1957–58 epidemic. For example, Henderson et al. [41] indicate that over 60% of students had clinical illnesses during the autumn of 1957 and that data from 28 U.S. school systems showed increases of 20% to 30% absenteeism above normal. The rates in New York City were a little higher, with school absenteeism reaching 29% of all school attendees and 43% for Manhattan.

This agent-based model was programmed in C++. Simulations were run at the Pittsburgh Supercomputing Center on its Blacklight architecture. As reported in this paper, each result is the average of 20 successfully initiated epidemics, each of which was run in parallel on a separate compute core (PC computer). Each simulation required 15 minutes.

## Results

Simulation results are illustrated by overall attack rate in the population defined the percentage of total population both infected *and* symptomatic (Table 3) for the five Types of school closure authority. At baseline with no school closures, influenza attack rates were 20% at R0 = 1.2, over 36% at R0 = 1.6, and 47% at R0 = 2.0. These represent the ranges of rates previously reported for seasonal influenza combining all population ages [43], compared to the high R0 of 2.35 reported by Earn et al. for the 2009 H1N1 pandemic for the school-age population alone [16]. For all Types and at all transmissibility levels, the attack rates among school children were higher than among adults (data not presented).

**Table 3:** Attack Rates* of Novel Influenza with Transmissibility of R0 = 1.2, 1.6, and 2.0 without School Closure (baseline) and under Five Types of State School Closure Authority

**School Closure Authority**	**Closure Threshold**	**Closure Duration**	Attack Rates *		
			**R0=1.2**	**R0=1.6**	**R0=2.0**
Baseline	None	None	20%	36%	47%
TYPE I	1% statewide prevalence, 1-day implementation	1 week	19%	36%	46%
		2 weeks	19%	36%	46%
		4 weeks	19%	35%	45%
		8 weeks	14%	33%	43%
		16 weeks	2%	27%	43%
TYPE I variation	0.1% statewide prevalence	8 weeks	16%	35%	43%
	10% statewide prevalence	8 weeks	12%	31%	44%
TYPE II	5 cases per school	1 week	19%	36%	47%
		2 weeks	19%	36%	46%
		4 weeks	19%	35%	44%
		8 weeks	14%	32%	43%
		16 weeks	2%	27%	43%
TYPE III	1% prevalence, 3-day delay	8 weeks	14%	32%	43%
	1% prevalence, 5-day delay	8 weeks	13%	31%	44%
	1% prevalence, 10-day delay	8 weeks	10%	33%	46%
TYPE IV	Random, 1–3 cases per school	8 weeks	16%	32%	43%
	Random, 1–5 cases per school	8 weeks	15%	32%	43%
	Random, 1–10 cases per school	8 weeks	16%	32%	43%
TYPE V	5 cases per school	Random, re-open 1–3 days before 8 weeks	15%	32%	43%
	5 cases per school	Random, re-open 1–10 days before 8 weeks	17%	33%	43%
	5 cases per school	Random, re-open 1–20 days before 8 weeks	18%	33%	43%
	5 cases per school	Random, re-open 1–30 days before 8 weeks	18%	33%	44%

*Infected and symptomatic individuals as a percentage of total population.

With uniform statewide closures in Type I, there was little effect on attack rates unless schools were closed for at least 8 weeks at any R0 tested. At low R0 of 1.2, an 8-week closure resulted in a 14% attack rate when triggered by a 1% statewide prevalence. At higher R0’s of 1.6 and 2.0, school closures of any duration had little effect on attack rates.

The Type I variations of prevalence threshold with low R0 of 1.2 produced counter-intuitive results: the lower statewide prevalence threshold of 0.1% had a higher (16%) attack rate; and the higher statewide prevalence threshold of 10% had a lower (12%) attack rate. These differences have been observed in our previously published school closure studies [8,19], and arise from shifting the school-closure period to later, thus having greater impact on the peak and duration of the epidemic curve. This also explains why the benefit of using a higher prevalence threshold disappears from the results at higher R0s, when the epidemic peak occurs earlier.

**Figure 1:**
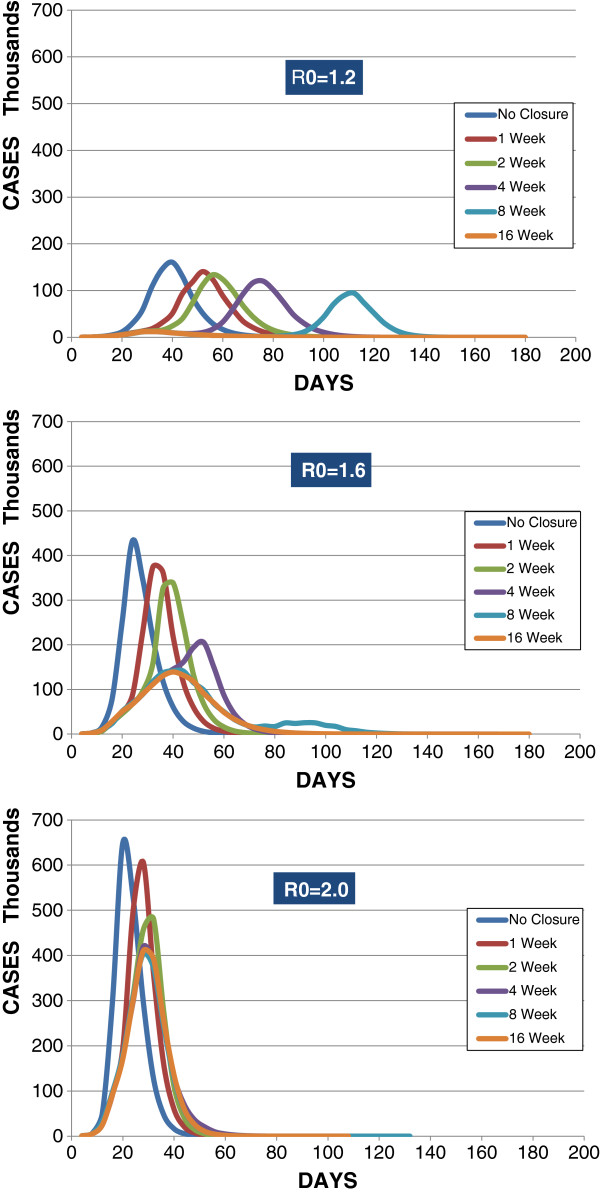
Type II - Cases per day with 5-case per-school closure threshold for varied durations at R0 = 1.2, 1.6, and 2.0.

With Type II using a local threshold (5 cases per school), attack rates resulting from closures of various durations were the same as those with statewide uniform threshold (1% prevalence). Figure 1 graphically shows Type II, which uses a 5-case per school trigger and 1-day implementation. For R0 = 1.2, the peak incidence is about 140,000 with 1 week closure; 134,000 with 2 weeks; 120,000 with 4 weeks; 94,500 with 8 weeks; and 12,000 with 16 weeks; the peak day is later for 1, 2, 4, and 8 weeks of closure (day 52, 56, 76, and 112, respectively) but earlier for 16 weeks of closure (day 32). For R0s of 1.6 and 2.0, the peak is lower at each successively longer duration of closure, but nearly all peaks are both earlier and higher than for the same closure durations at R0 = 1.2. At R0 = 1.6 the latest peak (day 52) occurs with a 4-week closure, when incidence of 205,000 is higher than with 8- and 16-week closures but well below incidences when closures are 2 weeks or less. At R0 = 2.0, transmission occurs so rapidly that the peak day occurs between 29 and 32 regardless of closure duration. Also at this high R0, increasing closure durations reduced the peak incidence successively from over 600,000 at 1 week, to 480,694 at 2 weeks; but beyond closure duration of 4 weeks with 419,644 cases, longer closures produced little further reduction of incidence, which remained above 400,000 cases with both 8- and 16-week closures.

Type III tested another approach to altering statewide uniformity of closure threshold: by delaying implementation, the effective prevalence of infections was allowed to rise before schools were actually closed. At R0 = 1.2, compared with the attack rate of 14% from 1-day implementation based on 1% statewide prevalence, 3-day delay made no difference (14% attack rate), 5-day delay brought improvement (13% attack rate), and 10-day delay brought even further improvement (10% attack rate). As with the Type I variation of prevalence threshold, these results arise from shifting the school-closure period to later in the epidemic. However, at higher R0s, the delay-related shifting of school closure period produced no benefit because of the epidemics’ earlier peaks.

Type IV shows the effect of deferring to local decision makers and locally determined closure thresholds. Here, with low R0 (1.2) virus transmissibility, allowing randomness among individual schools in the case number to trigger an 8-week closure resulted in attack rates little different than requiring a statewide trigger of 5 cases (14%): 1–3 day triggers had a 16% attack rate; 1–5 day triggers had a 15% attack rate; and 1–10 day triggers had a 15% attack rate. At the higher R0s, local randomness in closure triggers had no effect on attack rates, which remained at 43%—the same as with statewide 5-case triggers.

**Figure 2:**
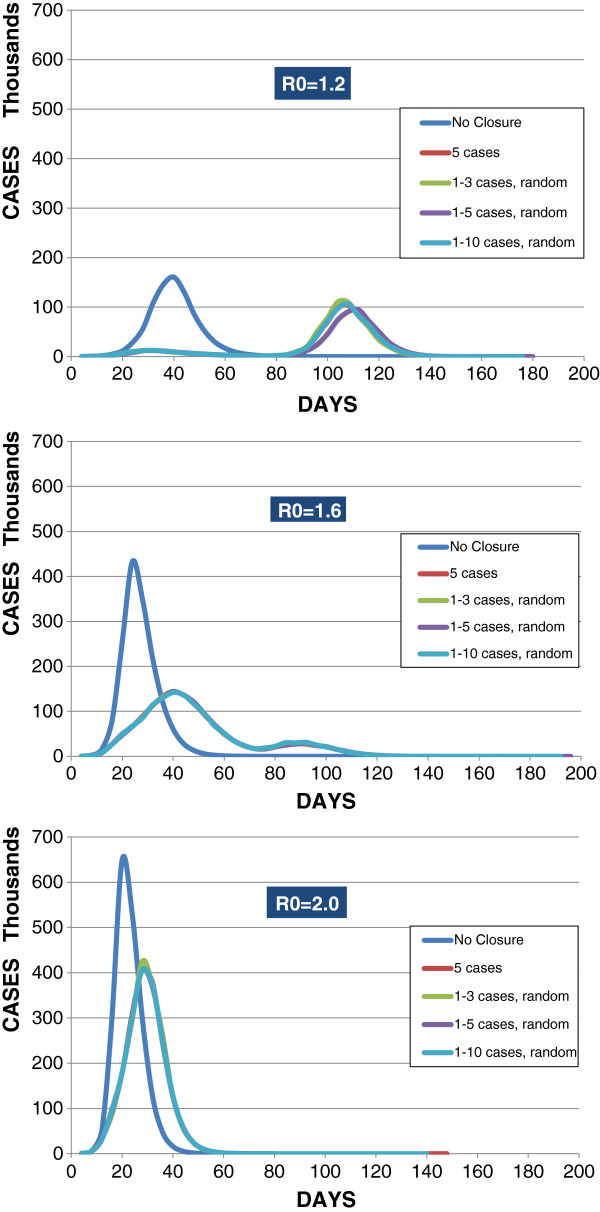
Type IV - Cases per day for 8-week closure duration with varied per-school closure thresholds at R0 = 1.2, 1.6, and 2.0.

Figure 2 shows how with Type IV the epidemic peaks varied as a function of changing the threshold for initiating an 8-week school closure. At all R0’s tested and regardless of whether the per-school case incidence threshold was uniform or randomized, the peak day and peak incidence were very similar. However, at R0 = 1.2, the random 1–5 case threshold produced a slightly later peak (day 108) than the alternatives (all at day 104); and notably, at R0 = 1.2 and 1.6, random thresholds (1–5 cases and 1–10 cases, respectively) produced slightly lower case incidences (91,000 cases and 142,000 cases, respectively) than using the uniform 5-case closure threshold (104,000 and 144,000 cases, respectively). At R0 = 2.0, using the 1–3 case random threshold resulted in higher incidence than the uniform and random threshold alternatives (425,286 cases compared to alternatives approximating 407,000 cases).

Type V shows how failure to sustain a school closure for the full 8 weeks for low R0 virus slightly erodes the benefit on attack rates. Compared with attack rate of 14% for full 8-week closure, the rates are 15% with 3-day premature re-opening, 17% with 10-day premature re-opening, and 18% with 20-day or 30-day premature re-opening.

**Figure 3:**
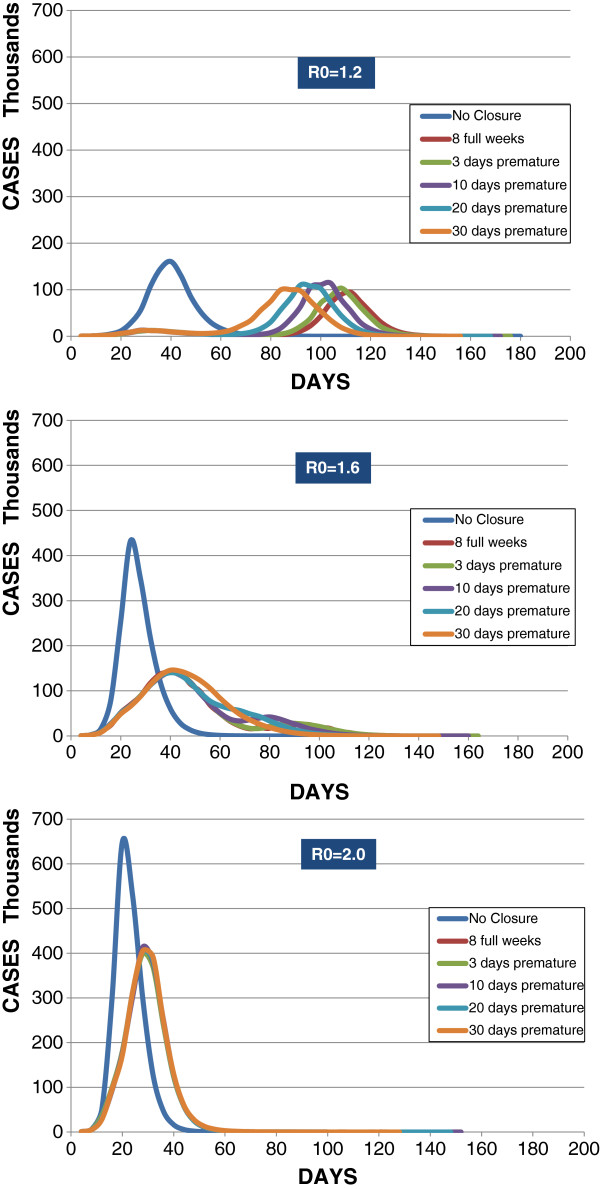
Type V - Cases per day with varied per-school re-openings before 8 weeks at R0 = 1.2, 1.6, and 2.0.

Figure 3 shows with Type V the effects of prematurely re-opening schools. The figure shows epidemic peaks when 8-week closures initiated by a 5-case per-school threshold are prematurely terminated by 3, 10, 20, and 30 days. At R0 = 1.2, premature re-openings produced successively earlier and higher peaks, except that the 30-day premature reopening had a lower, flatter peak incidence—raising attack rates to 18% (as shown on Table 1) and eroding the benefit of the 8-week closure period. At R0 = 1.6, the peak occurs at day 40 regardless of premature re-opening schedule, and the incidence varies only slightly among them (between 141,000 and 146,000). At R0 = 2.0, the peak occurs at day 28 regardless of premature re-opening, and the incidence again varies only slightly among them (between 399,000 and 406,000 cases).

For 8-week closures with R0s of 1.6 and 2.0, there is little or no change in peak day or incidence whether school closure threshold is standardized at 5-cases per school (Type II) or randomized at 1–3, 1–5, and 1–10 cases per school (Type IV) or whether there is premature re-opening (Type V).

## Discussion

These simulation experiments permit comparisons among the states’ various delegations of school closure authority and their variations in specificity for criteria determining the uniformity, timing, and duration of school closure as an influenza mitigation strategy.

On the question of whether centralized statewide authority is advantageous for epidemic mitigation, these results show no basis for justification. There is little difference in effectiveness as measured by attack rate whether school-closure is statewide or school-by-school. At low virus transmissibility (R0 = 1.2), the more important factor is the duration of closure: attack rates remain high unless closures last 8 weeks or more. When transmissibility of infection is high (R0 = 1.6 and 2.0), even differences in closure duration have little effect on attack rates. But transmissibility is difficult to discern in an ongoing epidemic; and lengthy closures impose social and economic burdens on communities and families. Therefore, the school closure decision might reasonably be devolved to local officials who understand, and are accountable for, health issues and socio-economic concerns of local populations.

School-specific closure criteria, though absent from most states’ existing epidemic influenza plans, appear to be important in disease control because of the dynamics of transmission. Influenza arrives in localities at different times, and commuting and mixing patterns vary locally [35,36]. Thus, even if the case-per-school closure threshold is non-uniform across school districts and communities, there is a disease-control benefit to local responsiveness. Again, these results do not outweigh the possible advantage of decision-making by knowledgeable local authorities.

On the question of prevalence threshold for school closure, these simulations offer no evidence to support school closures promptly at the earliest stage of an epidemic, given the need for an 8-week closure period and the typically unknown transmissibility of the virus during an epidemic. At low R0, delaying closures shifts the closure period further toward the epidemic peak. At higher R0s, this shift is irrelevant because the epidemic peak occurs so rapidly; and so closing schools at all has minimal effect. Again, since transmissibility is usually unknown during an epidemic, there may be an advantage to delaying school closures while monitoring increasing case numbers over a week or two at the individual community or school level.

An interesting observation concerns the timing of school closures. Earn *et al*. reported that school closure of 8 to 10 weeks in summer for the 2009 H1N1 pandemic in Alberta, Canada was followed by a spike in the fall infection rate (a so-called “second wave” of the pandemic) [16]. This effect also appears in our simulation results, when uniform, statewide closures initiated early in an epidemic delays the peak incidence but does not quench the epidemic.On the question of when to re-open, these results show that the duration of school closures affects not only attack rate but also peak day and peak incidence. Closures of 1–2 weeks or more, regardless of R0 or closure threshold, did not reduce attack rates but did delay the epidemic peak. Such brief closures offer the potential for reducing a likely surge in demand for healthcare services. Closures of less than 8 weeks may also allow time to use other influenza mitigation strategies such as distribution of vaccines and anti-viral medications.

These interpretations are influenced by recent experience of the H1N1 pandemic of 2009–10, which caused relatively mild illness. However, a more dangerous influenza virus might alter the implications of these results. When an influenza virus causes only mild disease, long-duration school closure may be economically and socially infeasible. But when influenza brings high rates of hospitalization and mortality, there may be weaker economic and social pressures against school closures. Then, longer school closure periods may have greater sustainability for households and workplaces as well as greater importance for disease control.

### Limitations

All computer models are simplifications of reality and can never account for every possible factor or interaction.

Each result presented represents an average of 20 simulation runs. This number is sufficient to demonstrate that the overall attack rates reported have only small variances at the model aggregate level. However, the distribution of more granular results, such as individual schools, would not demonstrate stability with 20 replicates.

This model explores in isolation the existing variations on school-closure authorities and specifications, though in reality this strategy might be combined with other social-distancing measures as well as pharmaceutical interventions such as vaccination.

## Conclusions

Decision makers need evidence on which to base school closure plans and procedures; but since controlled empirical studies of school closures are infeasible, computational modeling offers a useful alternative. A future influenza pandemic and its circumstances may not conform to the data and assumptions that our model drew from referenced sources or from previously published models. Thus, rather than dictate particular policy decisions, this model provides information to decision makers about possible results under a selected set of scenarios. It suggests ways to improve statutes and influenza plans by revealing the relative importance of criteria for decision making based on the competing priorities of epidemic mitigation and socio-economic advantage.

First, school closure should remain an optional strategy for influenza mitigation, given the uncertainties about virus transmissibility and disease severity that bedevil the early stages of an epidemic. Allowing for decentralized, local control over school-closure decisions may not affect attack rates and may improve responsiveness to local priorities and needs.

Second, the threshold for school closures need not necessarily be specified in states’ influenza plans, but provisions for early and ongoing *local* monitoring of case numbers should be well defined. It is important to avoid the risk contact-mixing among susceptible school children when the virus is still circulating widely—a situation more reliably observed at the community level rather than statewide.

Third, school closure authority may be exercised at the statewide or local level, so long as decisions are informed by monitoring incidence in local communities and schools. Local control may be important because of social and economic concerns about prolonged school closures, which local officials can weigh against the risks of disease perhaps more sensitively than statewide officials.

Fourth, depending on the transmissibility of an influenza virus, somewhat brief school closures of 1–2 weeks can help to reduce a surge in demand for healthcare services. Thus, school closures might be combined with other social-distancing strategies and with pharmaceutical interventions such as vaccinations.

## Competing interests

The authors declare that they have no competing interests.

## Authors’ contributions

MAP conceptualized this study, participated in design of modeling experiments, assisted in the interpretation of statutes and pandemic plans, and drafted the manuscript. STB participated in design of modeling experiments, developed the model, performed the modeling experiments, assisted in interpretation of the modeling results, and helped to draft the manuscript. PMS assisted in the conceptualization of the study, assisted with legal and policy data acquisition, created methodology for interpretation of statutes and pandemic plans, assisted in drafting of the manuscript. TBH participated in the study design, analyzed state school closure statutes and state pandemic plans, and helped to draft the manuscript. PCC developed the initial ABM, aided in model validations, and helped to draft the manuscript. SMG assisted in the conceptualization of the study design and helped to draft the manuscript. BYL contributed to the conceptualization and design of the model, interpretation of results, and drafting of the manuscript. CRK assisted in identifying data, participated in analysis, and edited the manuscript. JG contributed to the design of the model, the analysis and interpretation of the data, and helped to draft the manuscript. DSB made substantial contributions to conceptualizing how legal criteria could be used in a computational model and to the overall study design. All authors read and approved the final manuscript.

## Pre-publication history

The pre-publication history for this paper can be accessed here:

http://www.biomedcentral.com/1471-2458/12/977/prepub

## References

[B1] Glass LM Glass RJ Social contact networks for the spread of pandemic influenza in children and teenagers BMC Publ Health 2008 8 61 10.1186/1471-2458-8-61 PMC2277389 18275603

[B2] Mikolajczyk RT Akmatov MK Rastin S Kretzschmar M Social contacts of school children and the transmission of respiratory-spread pathogens Epidemiol Infect 2008 136 6 813 822 17634160 10.1017/S0950268807009181 PMC2870867

[B3] Hens N Goeyvaerts N Aerts M Shkedy Z Van Damme P Beutels P Mining social mixing patterns for infectious disease models based on a two-day population survey in Belgium BMC Infect Dis 2009 9 5 10.1186/1471-2334-9-5 19154612 PMC2656518

[B4] Koonin LM Cetron MS School closure to reduce influenza transmission Emerg Infect Dis 2009 15 1 137 138 author reply 138 10.3201/eid1501.081289 19116082 PMC2660715

[B5] Glezen WP Emerging infections: pandemic influenza Epidemiol Rev 1996 18 1 64 76 10.1093/oxfordjournals.epirev.a017917 8877331

[B6] Markel H Lipman HB Navarro JA Sloan A Michalsen JR Stern AM Cetron MS Nonpharmaceutical interventions implemented by US cities during the 1918–1919 influenza pandemic JAMA 2007 298 6 644 654 10.1001/jama.298.6.644 17684187

[B7] Halder N Kelso JK Milne GJ Developing guidelines for school closure interventions to be used during a future influenza pandemic BMC Infect Dis 2010 10 221 10.1186/1471-2334-10-221 20659348 PMC2915996

[B8] Lee BY Brown ST Cooley P Potter MA Wheaton WD Voorhees RE Stebbins S Grefenstette JJ Zimmer SM Zimmerman RK Simulating school closure strategies to mitigate an influenza epidemic J Public Health Man 2010 16 3 252 261 10.1097/PHH.0b013e3181ce594e PMC2901099 20035236

[B9] Human infection with new influenza A (H1N1) virus WHO Consultation on suspension of classes and restriction of mass gatherings to mitigate the impact of epidemics caused by influenza A (H1N1), May 2009 Releve epidemiologique hebdomadaire / Section d'hygiene du Secretariat de la Societe des Nations = Weekly epidemiological record / Health Section of the Secretariat of the League of Nations 2009 84 27 269 271 19575526

[B10] Update on school (K-12) and child care programs Interim CDC guidance in response to human infections with the novel influenza A (H1N1) virus [homepage on the internet] http://www.cdc.gov/h1n1flu/schools/

[B11] Hodge JG Bhattacharya D Gray J Legal preparedness for school closures in response to pandemic influenza and other emergencies 2008 The Center for Law and the Public's Health at Georgetown and Johns Hopkins Universities, A report submitted to the Centers for Disease Control and Prevention

[B12] Bell DM Non-pharmaceutical interventions for pandemic influenza, national and community measures Emerg Infect Dis 2006 12 1 88 94 16494723 10.3201/eid1201.051371 PMC3291415

[B13] H1N1 flu & U.S. Schools Answers to frequently asked questionshttp://www2.ed.gov/admins/lead/safety/emergencyplan/pandemic/guidance/flu-faqs.pdf

[B14] U.S. Department of Education 2009 Year in Review 2010 7 8 Accessed 11/13/12 at http://www2.ed.gov/about/reports/annual/2009review.html

[B15] Klaiman T Kraemer JD Stoto MA Variability in school closure decisions in response to 2009 H1N1: a qualitative systems improvement analysis BMC Publ Health 2011 11 73 10.1186/1471-2458-11-73 PMC3039590 21284865

[B16] Earn DJ He D Loeb MB Fonseca K Lee BE Dushoff J Effects of school closure on incidence of pandemic influenza in Alberta, Canada Ann Intern Med 2012 156 3 173 181 22312137 10.7326/0003-4819-156-3-201202070-00005

[B17] Sadique MZ Adams EJ Edmunds WJ Estimating the costs of school closure for mitigating an influenza pandemic BMC Publ Health 2008 8 135 10.1186/1471-2458-8-135 PMC2377259 18435855

[B18] Lempel H Epstein JM Hammond RA Economic cost and health care workforce effects of school closures in the U.S PLoS currents 2009 1 RRN1051 20025205 10.1371/currents.RRN1051 PMC2762813

[B19] Brown ST Tai JH Bailey RR Cooley PC Wheaton WD Potter MA Voorhees RE LeJeune M Grefenstette JJ Burke DS Would school closure for the 2009 H1N1 influenza epidemic have been worth the cost? a computational simulation of Pennsylvania BMC Publ Health 2011 11 353 10.1186/1471-2458-11-353 PMC3119163 21599920

[B20] Berkman BE Mitigating pandemic influenza: the ethics of implementing a school closure policy J Public Health Man 2008 14 4 372 378 10.1097/01.PHH.0000324566.72533.0b 18552649

[B21] Dalton CB Durrheim DN Conroy MA Likely impact of school and childcare closures on public health workforce during an influenza pandemic: a survey Commun Dis Intell 2008 32 2 261 262 18767427 10.33321/cdi.2008.32.26

[B22] Archived State Pandemic Influenza Plans [MIDAS Website] http://bit.ly/archivedpandemicfluplans

[B23] Cooley P Lee BY Brown S Cajka J Chasteen B Ganapathi L Stark JH Wheaton WD Wagener DK Burke DS Protecting health care workers: a pandemic simulation based on Allegheny County Influenza Other Respi Viruses 2010 4 2 61 72 10.1111/j.1750-2659.2009.00122.x 20167046 PMC2894576

[B24] Lee BY Brown ST Cooley PC Zimmerman RK Wheaton WD Zimmer SM Grefenstette JJ Assi TM Furphy TJ Wagener DK A computer simulation of employee vaccination to mitigate an influenza epidemic Am J Prev Med 2010 38 3 247 257 10.1016/j.amepre.2009.11.009 20042311 PMC2833347

[B25] Lee BY Brown ST Korch GW Cooley PC Zimmerman RK Wheaton WD Zimmer SM Grefenstette JJ Bailey RR Assi TM A computer simulation of vaccine prioritization, allocation, and rationing during the 2009 H1N1 influenza pandemic Vaccine 2010 28 31 4875 4879 10.1016/j.vaccine.2010.05.002 20483192 PMC2906666

[B26] Beckman RJ Baggerly K McKay M Creating synthetic baseline populations Transport Res A-Pol 1996 30 6 415 429

[B27] Public-Use Microdata Samples (PUMS) http://www.census.gov/main/www/pums.html

[B28] Common Core of Data: Build A Tablehttp://nces.ed.gov/ccd/bat/

[B29] Cajka JC Cooley PC Wheaton WD Attribute Assignment to a Synthetic Population in Support of Agent-Based Disease Modeling. RTI Press publication No. MR-0019-1009 2010 RTI International, Research Triangle Park, NC Retrieved [date] from http://www.rti.org/rtipress 10.3768/rtipress.2010.mr.0019.1009 PMC3347710 22577617

[B30] Census 2000 special tabulation Census tract of work by Census tract of residence (STP 64) http://www.census.gov/mp/www/cat/decennial_census_2000/census_2000_special_tabulation_census_tract_of_work_by_census_tract_of_residence_stp_64.html

[B31] Cooley P Brown S Cajka J Chasteen B Ganapathi L Grefenstette J Hollingsworth CR Lee BY Levine B Wheaton WD The role of subway travel in an influenza epidemic: a New York City simulation J Urban Health 2011 88 5 982 995 10.1007/s11524-011-9603-4 21826584 PMC3191213

[B32] Germann TC Kadau K Longini IM Jr Macken CA Mitigation strategies for pandemic influenza in the United States Proc Natl Acad Sci USA 2006 103 15 5935 5940 10.1073/pnas.0601266103 16585506 PMC1458676

[B33] Longini AN I Xu S Containing pandemic influenza at the source Science 2005 309 1083 1087 10.1126/science.1115717 16079251

[B34] Germann T Kadau K Longini IJ Macken C Mitigation strategies for Pandemic Influenza in the United States PNAS 2006 103 15 5935 5940 10.1073/pnas.0601266103 16585506 PMC1458676

[B35] Ferguson N Cummings D Cauchemez S Strategies for containing an emerging influenza pandemic in Southeast Asia Nature 2005 437 209 214 10.1038/nature04017 16079797

[B36] Ferguson NM Cummings DA Fraser C Cajka JC Cooley PC Burke DS Strategies for mitigating an influenza pandemic Nature 2006 442 7101 448 452 10.1038/nature04795 16642006 PMC7095311

[B37] Hens N Ayele GM Goeyvaerts N Aerts M Mossong J Edmunds JW Beutels P Estimating the impact of school closure on social mixing behaviour and the transmission of close contact infections in eight European countries BMC Infect Dis 2009 9 187 10.1186/1471-2334-9-187 19943919 PMC2799408

[B38] Aledort JE Lurie N Wasserman J Bozzette SA Non-pharmaceutical public health interventions for pandemic influenza: an evaluation of the evidence base BMC Publ Health 2007 7 208 10.1186/1471-2458-7-208 PMC2040158 17697389

[B39] Occupational Employment Statistics http://www.bls.gov/OES/Current/OES_Nat.htm

[B40] Cauchemez S Valleron AJ Boelle PY Flahault A Ferguson NM Estimating the impact of school closure on influenza transmission from Sentinel data Nature 2008 452 7188 750 754 10.1038/nature06732 18401408

[B41] Henderson DA Courtney B Inglesby TV Toner E Nuzzo JB Public health and medical responses to the 1957–58 influenza pandemic Biosecur Bioterror 2009 7 3 265 273 10.1089/bsp.2009.0729 19656012

[B42] Thanner MH Links JM Meltzer MI Scheulen JJ Kelen GD Understanding estimated worker absenteeism rates during an influenza pandemic Am J Disaster Med 2011 6 2 89 105 21678819

[B43] Basta NE Chao DL Halloran ME Matrajt L Longini IM Jr Strategies for pandemic and seasonal influenza vaccination of schoolchildren in the United States Am J Epidemiol 2009 170 6 679 686 10.1093/aje/kwp237 19679750 PMC2737588

